# A Swiss real world best practice experience in three different clinical settings of the 6 hour fingolimod first dose observation procedure

**DOI:** 10.1186/s40360-015-0006-0

**Published:** 2015-04-01

**Authors:** Simon P Ramseier, Serge Roth, Adam Czaplinski

**Affiliations:** Cantonal Hospital Aarau, Aarau, Switzerland; Clinique de Carouge SA, Carouge, Switzerland; Neurocentre Bellevue, Theaterstrasse 8, Zurich, CH-8001 Switzerland

**Keywords:** Multiple sclerosis, Fingolimod, Cardiovascular function, Heart rate

## Abstract

**Background:**

The Swiss label of oral fingolimod (0.5 mg once daily) requires a 6-hour first dose observation (FDO) including an ECG prior to and 6 hours after the first intake but in comparison to other countries such as Austria, Australia and Canada there are no restrictions regarding the clinical settings of the FDO procedure in Switzerland. We present here our real-world experience of the 6 hour FDO procedure in three different clinical settings, following fingolimod treatment initiation. This is the first report on the FDO of fingolimod in these real-world clinical settings in Swiss patients with multiple sclerosis (MS).

**Methods:**

This was a retrospective, multi-clinic, observational study of 136 patients with relapsing-remitting multiple sclerosis. Summary statistics have been used to present the data.

**Results:**

Only two patients (<1.5% [2/136]) experienced symptoms after the first dose of fingolimod. Atrioventricular conduction abnormalities were reported in 3% (4/136) of patients, which resolved spontaneously within 24 hours of treatment initiation. During the average 6.8 months follow-up, 96% (131/136) of the patients remained on therapy

**Conclusions:**

These findings support the safety and feasibility of FDO and tolerability of fingolimod in real-world clinical settings.

## Background

Fingolimod 0.5 mg once-daily (FTY720; Gilenya™, Novartis Pharma AG, Basel, Switzerland), a sphingosine 1-phosphate (S1P) receptor modulator, is the first oral therapy approved by the Swiss Regulatory Agency for treating patients with relapsing-remitting multiple sclerosis (RRMS) to reduce the frequency of relapses and delay disability progression [1]. Various pharmacodynamics effects of fingolimod are manifested as a consequence of the fingolimod mechanism of action of S1P receptor modulation since these receptors are ubiquitously distributed across different tissues [2]. Fingolimod initiation is associated with a transient reduction in heart rate and possible disturbances in atrioventricular (AV) conduction within the first few hours after first intake [3-5]. Hence, already at the time of market authorization in January 2011, the Swiss label of fingolimod made it a mandate to perform an ECG prior to and after 6 hours of the first dose administration, and recommended regular monitoring of blood pressure and pulse (first dose observation, FDO), which is similar to the current recommendations of other international health authorities. Here, we report for the first time the real-world experience of fingolimod treatment initiation and 6 hours FDO procedure in three different clinical settings outside of University Hospitals (MS centre, day clinic, private practice) since there are no restrictions on location of the FDO procedure in Switzerland.

## Methods

Data were collected retrospectively from the charts of RRMS patients treated and monitored as required by the Swiss label for fingolimod between August 2011 and May 2012 at three different locations (i.e. it did not encompass the new recommendations regarding the observation of patients with pre-existing cardiac conditions, published by the Swiss Regulatory Agency in October 2012 [1]). Site 1 was the MS centre, Cantonal Hospital Aarau, Aarau (*n* = 58), site 2 was the Clinique de Carouge in Carouge (office-based neurologist using a day clinic for FDO, *n* = 17) and site 3 was the Neurocentre Bellevue in Zurich, an office-based neurologist performing the FDO in his practice (*n* = 61). Prior to the FDO appointment all patients received essential information on fingolimod from their treating physician. They were informed about the potential side effects of fingolimod (short and long term), about the FDO procedure, including the reasons for ECG and the 6 h observation. Information was also provided on the required follow-up examinations after FDO over the next few months, including blood analysis and ophthalmological examination required by the Swiss label. Patients received recommendations on taking tablets including explanation of tablet packaging and drug description. The Cantonal Ethics Committee Zurich waived the review of this study as the data were obtained from retrospective chart reviews, and the information was recorded by the investigator in such manner that subjects cannot be identified, directly or through identifiers linked to the subjects.

## Results and discussion

### Overview of FDO process and associated workload

**Figure 1:**
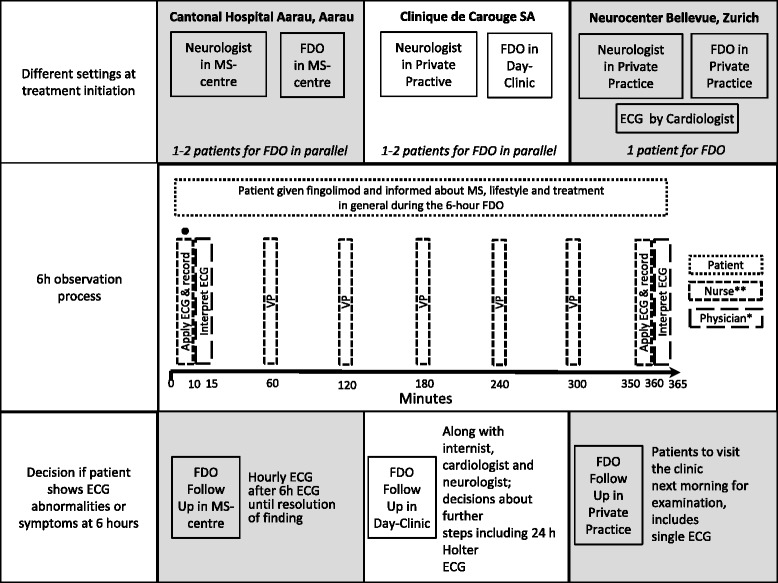
**Overview of the FDO process in the three different clinical settings.** Not for Neurocentre Bellevue. ECG recording was performed a number of days prior to FDO; *Depends on site, usually internist, cardiologist or neurologist; **Nurse or MS nurse; VP, vital parameters.

FDO measurements were performed in the daily clinical setting, which involved an ECG at the beginning and at the end of 6 hours and hourly recording of vital parameters (blood pressure and heart rate) (Figure 1). Between active FDO assessments, performed by the nurse or the physician, patients entertained themselves with activities such as reading, using their personal laptop, lunching together or discussing health related aspects of MS. A nurse took care of up to 2 patients using a single ECG device. She spent two times ten minutes to apply and record the ECG (prior to and 6 hours after the first intake), as well as five times 2 minutes to measure the vital parameters, representing a total workload of 30 minutes for the nurse over the 6 hour period. Interpretation of the ECG was carried out by a physician, representing a workload of 10 minutes altogether (two times five minutes for each ECG). The procedures upon appearance of ECG abnormalities or symptoms after 6 hours varied in the different clinical settings (see Figure 1). If, as stated in the Swiss label, heart rate dropped below 40 bpm during 6 hours FDO, another observation period of 6 hours (including ECG prior to and 6 hours after fingolimod administration) had to be performed on the second day of treatment.

### Real-world FDO outcomes in the three centres

Data was collected from 136 RRMS patients. 33 were treatment naïve and 103 were previously treated with interferon beta, glatiramer acetate or natalizumab. In total, 130 (95.5%) patients had uneventful FDO, 6 patients experienced cardiac events associated with the first dose (Table 1). Four patients had an AV block: 2 first-degree AV blocks and 2 second-degree AV blocks of Type Mobitz I. All the AV blocks detected resolved spontaneously within 24 hours. This was ensured either by monitoring with Holter ECG or an on-site ECG the following day. Two patients reported symptomatic events that resolved spontaneously without any pharmacological intervention (1 patient with vertigo-like sensation, 1 patient with palpitations [HR in normal range, 74 bpm]). The average duration of follow up was 6.8 months, and 131 (96%) of patients remained on therapy.

**Table 1:** **FDO outcomes in the three centres**

	**Site 1 Cantonal Hospital, Aarau**	**Site 2 Clinique de Carouge SA**	**Site 3 Neurocentre Bellevue**	**Total**
Total number of patients undergoing FDO	58	17	61	136
Patients with no FDO events (n)	57	16	57	130
Patients discharged at 6 hours (n)	57	16	59	132
Patients requiring extended observation after 6 hours (n)	1^a^	0	0	1
Patients requiring observation on 2nd day (n)	0	1^b^	2^c^	3
Symptomatic patients (n)	0	0	2^d^	2
Patients with ECG Abnormalities (n)	1^a^	1^b^	2^c^	4
1st degree AV Block (n)	0	0	2^c^	2
2nd degree AV Block Type I (Wenkebach) (n)	1^a^	1^b^	0	2
2nd degree AV Block Type II (Mobitz Type II) (n)	0	0	0	0
Symptomatic events that resolved by the end of 6 h observation (n)	0	0	2^d^	2
ECG events that had resolved at extended observation or follow-up examination on the 2nd day (n)	1^a^	1^b^	2^c^	4

^a^2nd degree AV block, Wenkebach type: extension of observation by 1 h and repeat of ECG; AV block had resolved.

^b^2nd degree AV block, Wenkebach type: 24 h Holter ECG as a precautionary measure; AV block had resolved and no further finding observed on Holter ECG.

^c^1st degree AV blocks: patients were asked to return to the practice the next day for a single ECG; AV block had resolved.

^d^1 patient with vertigo-like sensation, 1 patient with palpitations (HR in normal range 74 bpm): symptoms had resolved for both patients by the end of the 6 h observation.

AV, atrioventricular; HR, heart rate; bpm, beats per minute.

## Conclusions

The FDO experience reported here indicates that fingolimod is generally well tolerated upon treatment initiation. The majority of patients had no cardiac events during the FDO. Although symptomatic events were rare, the detection of 1st and 2nd degree Mobitz Type I AV blocks, which in some instances can have clinical implications, highlights the importance of monitoring the patients at treatment initiation and emphasizes the need for comprehensive information beforehand. All 3 participating sites capably facilitated the FDO procedure. Our data, which are in line with the phase 3 trial data [3,4] and other FDO related real-world observational studies [6,7], show that despite strict FDO guidelines in Switzerland, initiation of fingolimod therapy can also take place in clinical settings (MS centre, day clinic, private practice) outside of University Hospitals with a reasonable workload. They also support the safety and feasibility of FDO as well as the good tolerability profile of fingolimod in these real-world clinical settings, as shown by rates of adverse events and drop-outs comparable to those published previously [3,4], supporting the fact that fingolimod can safely be used in MS centres, day clinics and private practices.

## Abbreviations

S1P: Sphingosine 1-phosphate
RRMS: Relapsing-remitting multiple sclerosis
AV: Atrioventricular
FDO: First dose observation
ECG: Electrocardiogram
